# Hilab system, a new point-of-care hematology analyzer supported by the Internet of Things and Artificial Intelligence

**DOI:** 10.1038/s41598-022-13913-8

**Published:** 2022-06-21

**Authors:** Aléxia Thamara Gasparin, Claudiane Isabel Franco Araujo, Patricia Schmitt, Mônica Ribas Cardoso, Maiara Carolina Perussolo, Thainá Caroline Schuartz de Jesus, Erika Bergamo Santiago, Ivan Lucas Reis Silva, Ricardo Gurgel de Sousa, Flavia Zhu Teng, Evair Borges Severo, Victor Henrique Alves Ribeiro, Milena Andreuzo Cardoso, Fernanda D’Amico Silva, Carolina Rodrigues de Araujo Perazzoli, João Samuel de Holanda Farias, Bernardo Montesanti Machado de Almeida, Sergio Renato Rogal Júnior, Marcus Vinícius Mazega Figueredo

**Affiliations:** 1 Research and Development Department, Hilab, Hilab Campus, José A. Possebom, 800, Curitiba, Parana 81270-185 Brazil; 2 Erasto Gaertner Hospital, Ovande do Amaral, 201, Parana, Brazil

**Keywords:** Health care, Signs and symptoms, Diseases, Haematological diseases

## Abstract

The complete blood count (CBC) is one of the most requested tests by physicians. CBC tests, most realized in conventional hematological analyzers, are restricted to centralized laboratories due to frequent maintenance, large devices, and expensive costs required. On the other hand, most handheld CBC devices commercially available show high prices and are not liable to calibration or control procedures, which results in poor quality compared to standard hematology instruments. The Hilab system is a small-handed hematological platform that uses microscopy and chromatography techniques for blood cells and hematimetric parameters analysis through artificial intelligence, machine learning, and deep learning techniques. For clinical evaluation of the handheld CBC device, 450 blood samples were analyzed. The samples encompassed normal (82%) and pathological conditions (18%), such as thalassemias (2.2%), anemias (6.6%), and infections (9.2%). For all analytes, accuracy, precision, method comparison, and flagging capabilities of the Hilab System, were compared with the Sysmex XE-2100 (Sysmex, Japan) results. The sample source (venous and capillary) influences were also evaluated. Pearson correlation, Student *t* test, bias, and the Bland–Altman plot of each blood count analyte were calculated and shown. The significance level was set at p ≤ 0.05. For clinical evaluation, Hilab System and the Sysmex XE-2100 showed a strong correlation (r ≥ 0.9) for most evaluated parameters. In the precision study, analytes showed CV inside the limits established according to European Federation of Clinical Chemistry and Laboratory Medicine guidelines. The flagging capabilities of the Hilab system, compared to the manual microscopy technique, presented high sensibility, specificity, and accuracy. Venous and capillary samples (p > 0.05) do not differ statistically. Considering the need for point-of-care CBCs, the study indicated that the Hilab system provides fast, accurate, low cost, and robust analysis for reliable clinical use.

## Introduction

For providing important information about the general patient's health, the complete blood count (CBC) is one of the most ordered tests by physicians. Used as a screening, it is also essential for the diagnosis and evolutionary control of infectious diseases, medical emergencies, surgeries, and traumatology, in addition to chemotherapy and radiotherapy accompaniment^1^ and, therefore, considered an essential diagnostic tool by the World Health Organization^2^. The main parameters evaluated in CBC are the total count of red blood cells (RBC), platelets (PLT), and white blood cells (WBC). In addition, differential count of white blood cells and the determination of hematimetric parameters, such as hemoglobin (HB), hematocrit (HT), mean corpuscular volume (MCV) and mean corpuscular hemoglobin (MCH), is performed^3^.

Even considering that the gold standard method for cell identification is manual microscopy, mainly CBCs are realized in hematological analyzers, which use flow cytometry or resistivity-impedance methodologies^4^. However, these require frequent maintenance and are large and expensive devices, restricted to hospitals and central laboratories of considerable size. These features decrease the CBC’s access, especially to patients that live far from large urban centers^5^. In this sense, evaluating the availability of essential diagnostics tools like CBC in different countries on six continents, the authors suggest the existence of expressive gaps in diagnostic availability in diverse localities, especially in low and middle-income countries^6,7^. Besides, the CBC results take 24 h meantime, which usually promotes a delay in medical diagnosis. Thus, the development of hematology Point-Of-Care technologies (POCT) may allow greater access to exams and improved medical decisions^8^.

The Hilab system is a novel hematological platform that uses microscopy and chromatography techniques for blood cells and hematimetric parameters analysis. These small-handed devices are factory calibrated and combine artificial intelligence, machine learning, and deep learning techniques to provide the main parameters evaluated in the CBC test and four-part differential WBC. Simple operation single-use test kits accompany the Hilab System, which accepts venous or capillary samples (containing or not K3EDTA). Thus, this study evaluated the accuracy, flagging capabilities, precision studies, and the sample source influence of this new POCT, compared to the CBC results provided by Sysmex XE-2100 (Sysmex Corporation, Japan).

## Methods

### Sample preparation process

**Figure 1:**
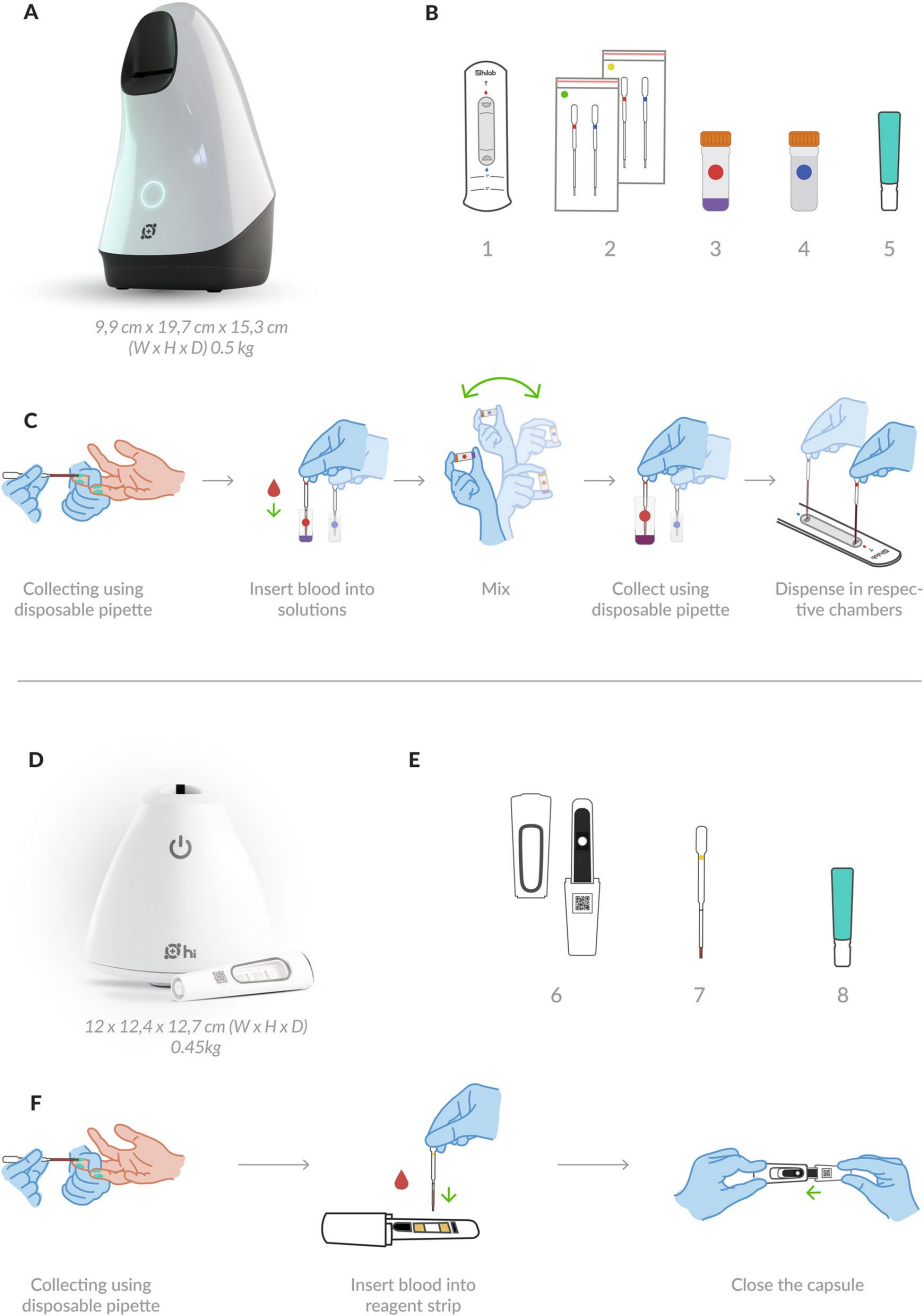

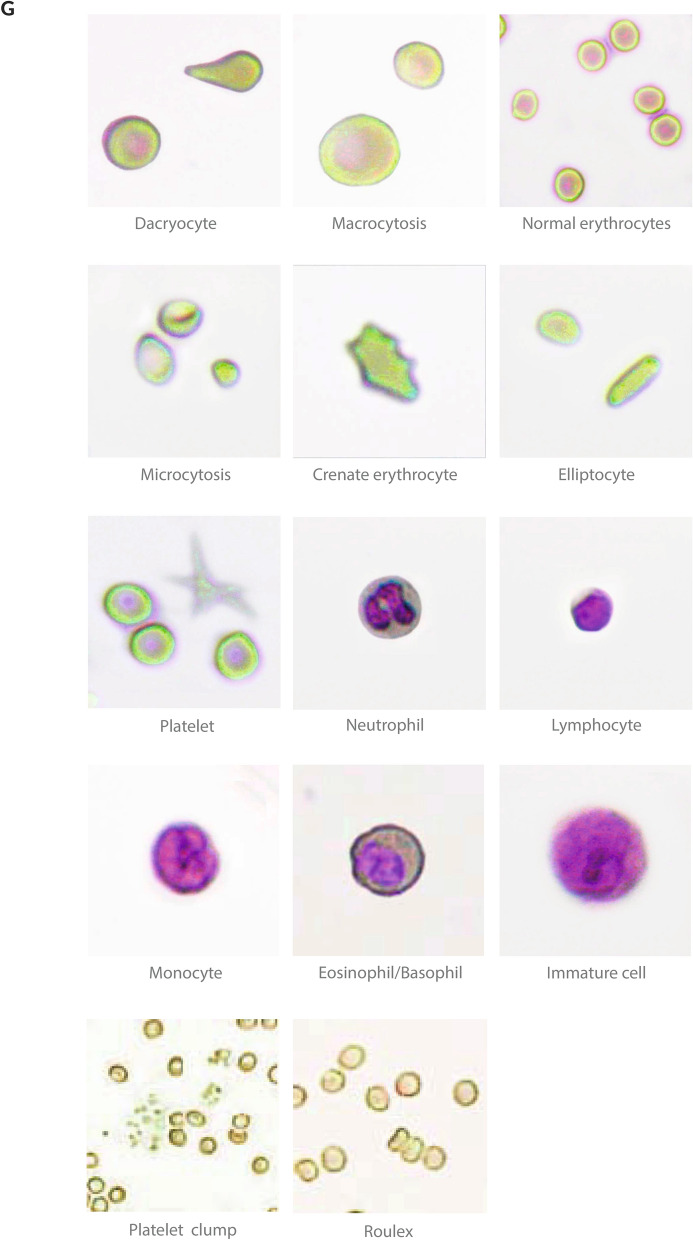
The Hilab system POCT hematology analyzer. Panel (**A**) shows Hilab lens device representation (Hilab, Brazil). (**B**) Components of the Hilab Lens test kit: (1) Hemocytometer, (2) Blood collection pipettes and Blood transfer pipettes, (3) Mixing-bottle of diluent 1; (4) Mixing-bottle of diluent 2; (5) Lancet. (**C**) Hilab Lens sample preparation workflow for capillary samples. Panel (**D**) shows the Hilab Flow device representation (Hilab, Brazil). (**E**) Components of the Hilab Flow test kit: (6) Capsule; (7) Blood collection pipette; (8) Lancet. (**F**) Hilab Flow sample preparation workflow for capillary samples. Both single-use test kits accompany isopropyl alcohol swab and curative. (**G**) Hilab’s software examples of evaluated blood cells.

The Hilab device accepts both venous and fingerstick blood samples. Two drops of blood, totaling 90 μl, are necessary for test realization. The sample is collected directly from the finger (or K_3_EDTA tube to venous workflow), using the components provided in the test kits. Two single-use diagnostic kits are utilized for the realization of point-of-care CBC tests. The first, used for cell counting, presents a disposable hemocytometer, the diluent solutions, blood collection pipettes, and blood transference pipettes. The second, used for hematimetric parameters evaluation, contains a chromatographic strip and a blood collection pipette (Fig. 1B,E, respectively). Both diagnostic kits present the materials needed for the capillary puncture.

#### Blood cell count

Two diluent solutions are needed to dye the cells and dilute the sample for blood cell counting. During the sample preparation, the test operator collects a drop of blood (40 uL) and places it into solution 1 (9-fold blood dilution). Solution 2 goes through the same process (179-fold blood dilution). After the blood homogenization, the test operator individually transfers a drop (10 μl) of solutions 1 and 2 to the hemocytometer chambers (Fig. 1C). The hemocytometer insertion into the Hilab Lens device is the last operation step.

The composition of the first diluent solution includes dyes, salts, and surfactants that promote RBC lyse and WBC differential stain. On the other hand, the second one is composed of different salts, which keep the natural morphology of RBC and PLT. Both solutions were developed and patented by Hilab^®^ and allow the liquid medium blood cells observation by optical microscopy.

#### Hematimetric parameters evaluation

For hematimetric parameters evaluation, a single drop of blood (10 μl) is collected and deposited on the chromatographic strip contained in a plastic capsule. This strip presents a fixed reagent that promotes RBC lysis and HB conversion into methemoglobin. The process is concluded with the insertion of the capsule into the Hilab Flow device for HB quantification (Fig. 1F). In this step, the test operator does not realize blood dilution. From obtained HB and RBC values, the artificial intelligence (A.I) estimates the HT, MCV, and MCH, based on previous studies (FAILACE and FERNANDES, 2015). The principle of these calculations is the correlation between HB and/or RBC values, with HT, MCV, and MCH values, allowing the estimation of these analytes.

### The device

The Hilab system uses microscopy and chromatography techniques to supply the CBC result. The microscopy one is handled by a small handheld device (19.7 × 9.9 × 15.3 cm; 0.5 kg) called Hilab Lens (National Health Surveillance Agency (ANVISA) registration no. 80583710018; Fig. 1A). The acquisition process occurs by the autofocus and image capture process, which takes upwards of 400 images of each sample to form the final image by the composition of all figures stacked. This device processes the blood sample in two stages: the first is used to read the WBC (first chamber), and the second is to read RBC and PLT (second chamber). Considering the standard dilution factor used in cell counting single-use kits, the number of cells that this device analyzes in each sample is dependent on the blood cell number of the patient. The chromatography technique is handled by a small handheld analyzer (12.4 × 12.4 × 12.7 cm; 0.45 kg) called Hilab Flow (ANVISA registration no. 80583710007; Fig. 1D). This device incorporates a camera-equipped light detector and sample integrated capsules that enable the processing of several analytes by the optical density of chromatography strips.

Through the calibration capsule, the test operator calibrates both devices every 24 h to verify the correct functioning of the sensors and the position of the focus mechanism. Hilab Flow and Lens need energy access for battery charging and an internet connection to enable the Hilab patient registration platform access. The patient registration data are encrypted and linked to the device and the exam QR code through a key. After sample processing, the Hilab System sends the patient data, test realization device, and the test operator register to the Hilab Software discussed below. Hilab uses two non-relational databases to protect the patient data and test results, precluding the information correlation in case of a system invasion.

### Imaging processing and object classification

For Hilab Lens, blood cell images go through a deep learning approach for both cell detection and classification. We applied data augmentation to RBC, PLT, and WBC subpopulations, generating more data through rotating and mirroring existing images. For cell detection, given that a single image covers thousands of cells—small objects—the image is divided into a grid and each sub-image is processed individually through a pipeline with the YOLO (You Only Look Once) deep learning approach, a state-of-the-art real-time object detection system that analyzes the image globally only once, using features from the entire image, to predict each bounding box (a box containing positions delimiting the detected object). The algorithm used for this analysis includes an overlap verification, in which detections that have less than a threshold of intersection are considered the same cell. Since YOLO also predicts class probabilities for each detected object, the network is also used for pipeline classification through the probability evaluation that a given region contains a specific type of object.

For hematimetric parameters, the Hilab Flow uses A.I. and computer vision methods for HB detection on the chromatographic strip. Thus, after the device collects the signals of the blood sample, signal processing techniques extract the sample mean colorimetric value, and the system applies regression analysis to obtain the HB concentration. Finally, from RBC and HB results, the Hilab System estimates the HCT, MCV, and MCH.

### Processing of results

After A.I image processing, Hilab’s software receives the figures through the internet connection. The software is an interactive platform developed by Hilab to connect the habilitated health professional to the A.I processed image of the blood sample. Only our trained professionals, with active registration in the professional class council, have access to this platform in which the professional double-checks the A.I results of blood samples and makes corrections, if necessary. If any divergence occurs during the exam analysis, the result provided by the habilitated professional is always prevalent about A.I.

The number of blood cells is obtained through the cell count considering the double-checked processed image, the standard dilution factor used in cell counting single-use kits (179-fold blood dilution for erythrocytes and platelets; 9-fold blood dilution for leukocytes), and the standard volume of the high precision hemocytometer chamber. Finally, the health professional issues the report, and the patient receives it signed by Hilab technical responsible and the analyst of the report, through email or SMS. Thus, the A.I accuracy guarantees fast analysis, and the specialists double-check results ensures a reliable exam report. The sample preparation, imaging acquisition, and processing of results by the Hilab system take an average time of 30 min. Through the Hilab software the health professional has access to the exam lot, test realization device, test operator register, and patient exam history. Hilab guarantees 24-7 service to the correct system function, reports delivery, and customer support.

### Clinical protocol

#### Method comparison

The Research Ethics Committee of the Paranaense League Against Cancer (CAAE; no. 49961421.3.1001.0098) approved this study, and all performed methods were in accordance with the relevant guidelines and regulations. Venous whole blood clinical samples (N = 450) were collected from patients aged between 0.6 and 86 years old, including males (42%) and females (58%), by trained and qualified professionals. All subjects or their legal guardian(s) informed consent for study participation. The samples encompassed normal (82%) and pathological conditions (18%), such as thalassemias (2.2%), anemias (6.6%), and infections (9.2%). The venous whole blood samples were stored in standard K3EDTA collection tubes (Vacuette^®^, Greiner Bio-One, Brazil) and processed within 12 h of collection. The sample processing included blood analysis in the Hilab system and the standardized Sysmex XE-2100 analyzer (Sysmex Corporation, Japan; reference values). Although traditional flow cytometry is considered the gold standard method for population cell differentiation, mainly due to the cost, most clinical laboratories use devices with the impedance-resistivity methodology for CBC realization, justifying the analyzer choice. The analyses of the Hilab System and Sysmex XE-2100 analyzer results were done by different professionals, being a double-blinded study.

Pearson correlation, Student *t* test, bias, and the Bland–Altman plot of each blood count analyte were calculated and shown. Also, we compared the Hilab System accuracy of each CBC analyte to the Sysmex XE-2100. Thus, we assessed each value inside (1) or outside (0) of the reference range through the confusion matrix. The evaluated parameters were specificity, sensitivity, kappa coefficient, and balanced accuracy. All biological samples collected were single-use for this study and discarded after the analysis, following the potentially infected samples standard procedure.

#### Precision study

K3EDTA whole blood venous samples were used for precision study due to the incompatibility of commercial hematological controls with the Hilab solutions. To encompass the different clinical ranges of CBC analytes, four extended ranges of each PLT, WBC, RBC, and HB sample, were measured ten consecutive times in three devices. For the repeatability study, within-day precision was evaluated by each range standard deviation (SD) and within coefficient of variation (CV). For the reproducibility study, this protocol was performed by two different operators and evaluated for three consecutive days. Similarly, the SD and CV evaluations of each clinical range were done.

#### Equivalence between capillary and venous samples

Fresh fingerstick blood samples were collected from healthy volunteers (n = 150) parallelly with venous blood collection. For all cell blood counts, results from capillary samples were compared with the respective venous plus anticoagulant (K_3_EDTA) samples using the Passing–Bablok analysis and paired Student *t* test.

#### Flagging study

The flagging capabilities of the Hilab system were compared to the manual microscopy technique, sending the blood smears to the analysis of trained personnel from a support laboratory (Diagnostico do Brasil^®^, Parana, Brazil). The evaluation of RBC morphological abnormalities, including microcytosis, anisocytosis, and macrocytosis, was realized. We also evaluated the PLT and WBC abnormalities, assessing the presence of platelet clumps and immature cells, respectively. The accuracy, specificity, sensibility, kappa coefficient, and balanced accuracy of each parameter were calculated and expressed.

### Statistical analysis

All data were analyzed and plotted using the R software statistics package analysis. The Kolmogorov–Smirnov normality test was applied to ensure that the data met the criteria for performing the parametric tests. CV, SD, Bias, Student *t* test, Bland–Altman, and Passing–Bablok analysis were calculated using this package. The significance level was set at p ≤ 0.05.

### Ethics approval and consent to participate

This study was approved by the Research Ethics Committee of the Paranaense League Against Cancer (CAAE no. 49961421.3.1001.0098).

## Results

### Method comparison

**Figure 2:**
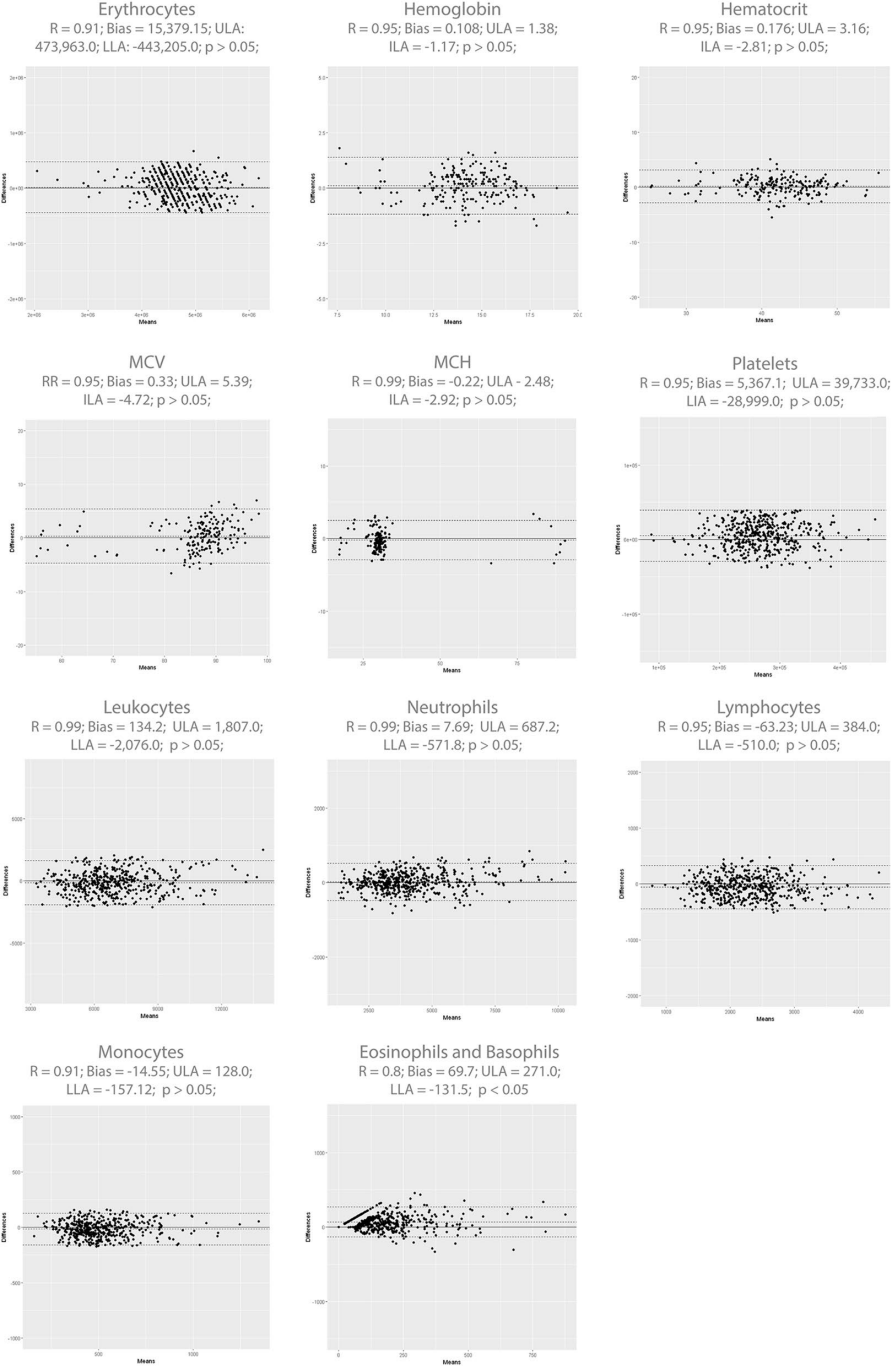
Bland–Altman plot of the method comparison study between the Hilab System and the Sysmex XE-2100. Pearson correlation, bias, Student *t* test p-value; upper limit of agreement (ULA), and lower limit of agreement (LLA) are demonstrated for each analyte.

The comparability study of CBC analytes is shown in Fig. 2 and Fig. S1. A wide range of values were evaluated for RBC (1.89–6.23 × 10^6^/mm^3^), HB (8.5–18.9 g/dL), HT (25.3–54.5%), MCV (61.8–96.1 fL), MCH (17.6–33.4 pg), PLT (91.0–571.0 × 10^3^/mm^3^), WBC (2.6–131.8 × 10^3^/mm^3^), neutrophils (NEU; 1.18–58.4 × 10^3^/mm^3^), monocytes (MON; 0.12–1.4 × 10^3^/mm^3^), lymphocytes (LINF; 0.77–8.7 × 10^3^/mm^3^), and eosinophils/basophils (EOS/BAS; 0–16.5 × 10^3^/mm^3^). As result, a high correlation (r ≥ 0.8) between the Hilab system and the Sysmex XE-2100 analyzer was observed for all analytes (r values; RBC—0.91; HB—0.95; HT—0.96; MCV—0.95; MCH—0.91; PLT—0.95; WBC—0.99; NEU—0.99; LIN—0.95; MON—0.91; EOS/BAS—0.8). Excepting EOS/BAS count (p < 0.05), for all analytes evaluated the results provided by methodologies were not statistically different (p < 0.05) each other.

The accuracy, specificity, sensibility, kappa coefficient, and balanced accuracy of each CBC analyte is shown in Table 1. The normal clinical range of RBC (Female (F): 3.9–5.1 × 10^6^/mm^3^; Male (M): 4.4–5.8 × 10^6^/mm^3^), HB (F: 11.3–15.1 g/dl; M: 12.3–16.9 g/dl), HT (F: 35.1–46.7%; M: 38–52.1%), MCV (F: 81–100.7 fl; M: 81. 5–101.8 fl), MCH (F: 26.3–32.6 pg; M: 26.9–33.1 pg), PLT (F: 126.6–344.7 × 10^3^/mm^3^; M: 128.4–302.1 × 10^3^/mm^3^), WBC (F: 2.9–10.05 × 10^3^/mm^3^; M: 2.8–9.7 × 10^3^/mm^3^), NEU (F: 0.590–6.5 × 10^3^/mm^3^; M: 0.550–6.35 × 10^3^/mm^3^), MON (F: 0.019–0.7 × 10^3^/mm^3^; M: 0.002–0.845 × 10^3^/mm^3^), LINF (F: 0.716–3.4 × 10^3^/mm^3^; M: 0.582–3.4 × 10^3^/mm^3^), and EOS/BAS (F: 0–0.574 × 10^3^/mm^3^; M: 0–0.718 × 10^3^/mm^3^) were established according previous studies^9^. As result, all analytes presented high values (≥ 0.8) of accuracy, specificity, sensibility and balanced accuracy. Also, all kappa coefficients were above 0.8 (RBC—0.94; HB—0.96; HT—0.94; MCV—0.89; MCH—0.89; PLT—0.95; WBC—0.89; NEU—0.95; LIN—0.86; MON—0.95; EOS/BAS—0.81).

**Table 1:** Accuracy, specificity, sensibility, kappa coefficient, and balanced accuracy of the method comparison study, comparing the Hilab system to Sysmex XE-2100.

	Accuracy (%)	Specificity (%)	Sensibility (%)	Kappa	Balanced accuracy (%)
RBC	99.3	93.0	99.7	0.94	96.2
HB	99.1	99.7	100.0	0.96	99.3
HT	98.7	96.3	98.9	0.94	97.6
MCV	97.6	88.5	98.9	0.89	93.7
MCH	97.0	93.6	97.6	0.89	95.6
PLT	99.8	99.9	99.8	0.95	99.0
WBC	98.0	93.5	98.6	0.89	96.0
NEU	99.4	96.5	99.6	0.95	98.07
MON	99.8	99.9	99.9	0.95	99.9
LINF	99.6	99.9	99.6	0.86	99.8
EOS/BAS	80.0	99.0	98.0	0.81	89.1

### Precision study

The repeatability and reproducibility studies performed using relevant clinical ranges of PLT, RBC, WBC, and HB, are shown in Table 2. According to European Federation of Clinical Chemistry and Laboratory Medicine guidelines (EFLM; CV variation; PLT < 10%; RBC < 4%; HB < 3.6%; WBC < 15.9%), all parameters presented the CVs inside the established limits (mean CV; RBC—3.08%; WBC—6.97%; PLT—8.16%; HB—1.63%). The estimated parameters (HT, MCV, and MCH), and the 4-part differential WBC analytes were not evaluated.

**Table 2:** Hilab system precision study. Coefficient of variation (CV) and standard deviation (SD) of each range are demonstrated.

	Target range	Repeatability study		Reproducibility study	
		CV (%)	SD	CV (%)	SD
RBC	3.0–4.0 × 10^6^/mm^3^	4.15	0.152 × 10^6^	4.10	0.148 × 10^6^
	4.0–4.5 × 10^6^/mm^3^	0.32	0.020 × 10^6^	2.77	0.131 × 10^6^
	4.0–5.0 × 10^6^/mm^3^	3.61	0.245 × 10^6^	2.99	0.192 × 10^6^
	5.0–6.0 × 10^6^/mm^3^	3.04	0.156 × 10^6^	3.70	0.390 × 10^6^
WBC	1.0–2.5 × 10^3^/mm^3^	10.97	0.306 × 10^3^	9.33	0.307 × 10^3^
	2.5–4.5 × 10^3^/mm^3^	5.64	0.412 × 10^3^	5.83	0.200 × 10^3^
	4.5–6.0 × 10^3^/mm^3^	5.38	0.225 × 10^3^	6.55	0.449 × 10^3^
	6.0–7.5 × 10^3^/mm^3^	6.90	0.278 × 10^3^	5.17	0.244 × 10^3^
PLT	50–90 × 10^3^/mm^3^	7.24	7.0 × 10^3^	7.70	10.5 × 10^3^
	90–150 × 10^3^/mm^3^	5.78	20.5 × 10^3^	8.93	20.3 × 10^3^
	150–250 × 10^3^/mm^3^	6.50	21.9 × 10^3^	8.80	24.3 × 10^3^
	250–400 × 10^3^/mm^3^	8.66	19.15 × 10^3^	11.70	20.2 × 10^3^
HB	4.0–8.0 g/dL	1.3	0.206	2.0	0.215
	8.1–12.9 g/dL	2.1	0.395	1.7	0.301
	13.0–15.9 g/dL	1.6	0.289	1.0	0.190
	16.0–19.0 g/dL	1.9	0.277	1.5	0.310

### Anticoagulant influence and the effect of sample type

**Figure 3:**
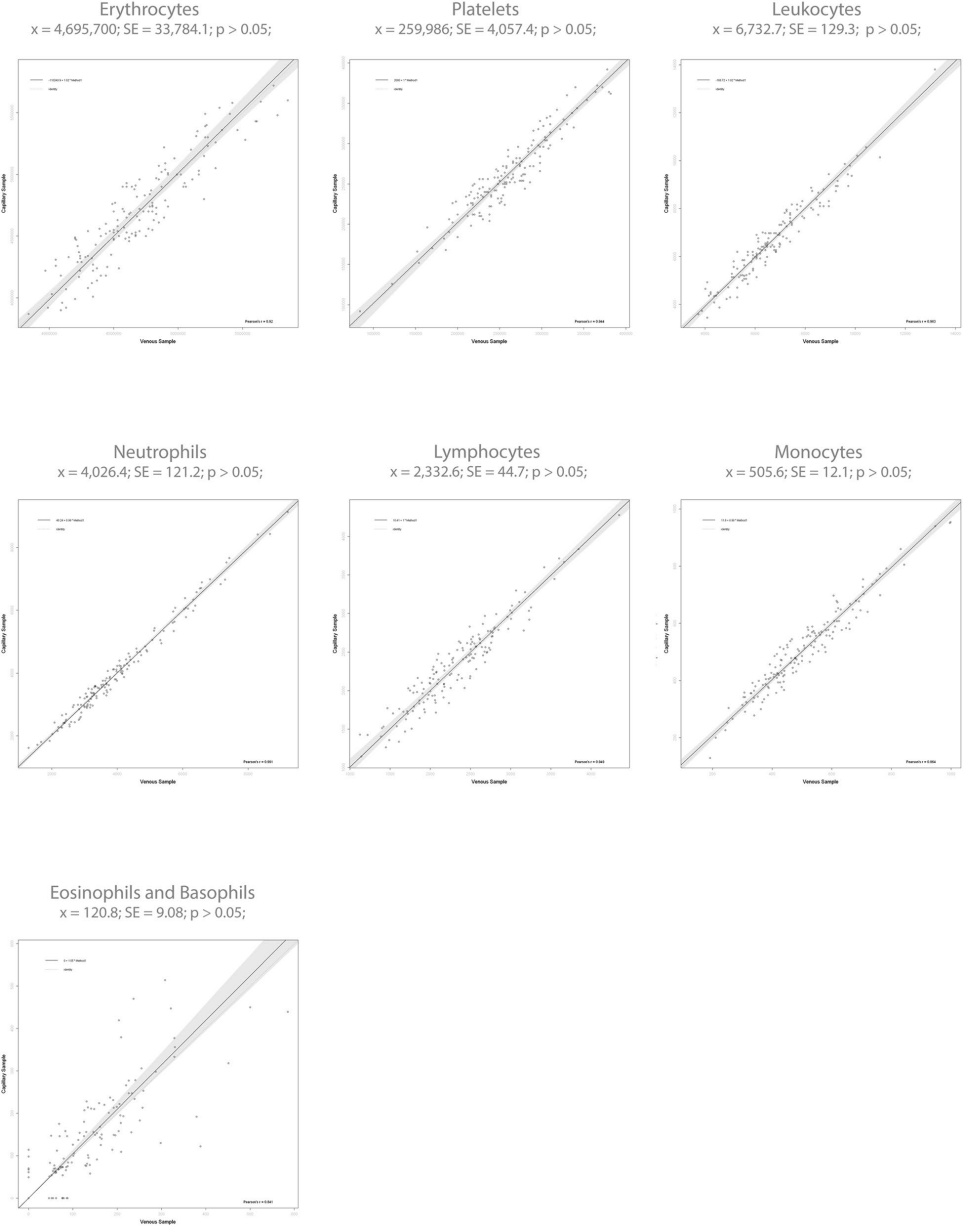
Anticoagulant influence and the effect of sample type. Graphs indicate the Hilab system results for venous (plus K_3_EDTA) × fingerstick blood samples. Mean (${\overline{\text{x}}}$), standard deviation (SD), and p-values of Paired Student *t* test are demonstrated for each analyte.

To assess the anticoagulant influence and the effect of sample type for cell analytes, the Hilab system results of whole venous blood were compared with the respective freshly fingerstick blood results (Fig. 3). Evaluating the paired Student *t* test, for all analytes, no statistical differences (p > 0.05) between venous (plus anticoagulant) and capillary blood samples were observed (p values; RBC—0.90; PLT—0.39; WBC—0.76; NEU—0.51; LIN—0.92; MON—0.60; EOS/BAS—0.67). Besides, all analytes presented high correlation (r ≥ 0.84) between venous and capillary blood samples (r values; RBC—0.92; PLT—0.94; WBC—0.96; NEU—0.99; LIN—0.95; MON—0.96; EOS/BAS—0.84).

### Flagging study

For RBC morphological abnormalities, 54 positive and 396 negative samples were analyzed. These analyses covered microcytosis, anisocytosis, and macrocytosis (Fig. 1G). As a result, high accuracy (97.06%), sensitivity (97.06%), specificity (100.0%), and balanced accuracy (98.1%) were observed. For PLT clumps, 17 positive and 433 negative samples were analyzed (Fig. 1G). Even with a minor number of positive samples, perfect accuracy (100.0%), sensitivity (100.0%), specificity (100.0%), and balanced accuracy were observed (100.0%). Finally, evaluating the presence of immature cells from both red and white lineage, 10 positive and 440 negative samples were analyzed (Fig. 1G). As shown above, high accuracy (95.73%), sensitivity (95.73%), specificity (100%), and balanced accuracy (89.9%) were observed (Table 3). Also, all evaluated parameters demonstrated strong kappa coefficients (RBC morphology—0.98; PLT—1.00; Immature cells—0.89).

**Table 3:** Accuracy, specificity, sensibility, kappa coefficient, and balanced accuracy of the flagging study, comparing the Hilab system to the microscopy technique.

Disturbance	Accuracy (%)	Specificity (%)	Sensitivity (%)	Kappa	Balanced accuracy (%)
RBC morphology	97.06	100.0	97.06	0.98	98.1
PLT	100.0	100.0	100.0	1.00	100.0
Immature cells	95.73	100.0	95.73	0.89	89.9

## Discussion

Most handheld CBC devices present high costs and are not liable to calibration or control procedures, which results in poor quality compared to standard instruments^10,11^. Our study provided an extensive clinical validation of the Hilab system to CBC point-of-care test, evaluating parameters like comparability, precision, and flagging studies.

The comparability study encompassed a significant range of values for all analytes, evaluating different health conditions. The sturdy values provided by the Hilab system, compared to the conventional hematological analyzer, can be observed by high Pearson correlation values (≥ 0.8), low bias, and the absence of statistically significant differences (p > 0.05) to most analytes (Fig. 2). The kappa coefficient results emphasize the reliable results of the Hilab System (≥ 0.8; Table 1), demonstrating a strong level of data agreement between these two different CBC methodologies, such as the strong values (≥ 0.8) of accuracy, specificity, sensitivity, and balanced accuracy. Besides, considering that changes in reference values of one or more analytes usually predict disease conditions, these results show that the Hilab System and Sysmex XE-2100 analyzer can similarly predict situations like these.

The EOS/BAS count was the only parameter that demonstrated differences statistically significant (p < 0.05) between Sysmex XE-2100 and Hilab results. Considering that the EOS and BAS are the less prevalent WBC subpopulation, the lower evaluation area (1 mm^2^) of the Hilab Lens device compared to Sysmex XE-2100 may influence these cell counts. In this sense, previous studies^12^ demonstrate that even considering conventional hematological analyzers, the slightest area evaluation differences result in differences in EOS and BAS quantifications. However, it's important to emphasize that considering the clinical range of these analytes, great values of accuracy, sensibility, specificity, and balanced accuracy were acquired (≥ 0.8; Table 1) with the Hilab system, as well as the kappa coefficient (> 0.8).

A special note should be taken regarding WBCs differentiation into four subpopulations (NEU, LINF, MONO, and EOS/BAS). Although other CBC devices provide 5-part WBC differentiation, the Hilab system can supply detailed patient health information, considering that 3-part hematology analyzers already provide enough information for most clinical settings. Also, in case of EOS/BAS count increase, the clinical report of patients can easily distinguish which cell subpopulation is changed.

The precision assay showed that all RBC, WBC, PLT, and HB levels presented the CV and SD values within the limits proposed by ELFM (Table 2). These data demonstrate the high repeatability and reproducibility of the Hilab system. Regarding other CBC analytes, in this assay, it was chosen not to evaluate the 4-part differential WBC analytes based on the incompatibility between commercial hematological controls and the Hilab reagents. Also, as HT, MCV, and MCH are estimated by HB and RBC values, these parameters were not regarded in this analysis.

As previously observed^5^, comparing the results of blood samples collected from venous (plus K_3_EDTA anticoagulant) or by finger stick, none cell analytes presented statistically significant differences (p > 0.05; paired *t* test; Fig. 3) among the collection methods. Therefore, the blood collection method of the Hilab system was validated. Furthermore, this data demonstrated that the anticoagulant K_3_EDTA does not interfere with the Hilab system result. Although hematimetric parameters were not evaluated in this analysis, other authors have already shown no statistically significant differences between venous and capillary samples for these analytes^13^. Also, tests demonstrated the absence of K_3_EDTA interference in HB results (data not shown).

Considering that the Hilab System uses the microscopy technique, considered the gold standard method for cell identification, the flagging study focused on the main CBC test alterations analysis: RBC morphological variation, PLT clumps, and the presence of immature cells. For all alterations evaluated (Table 3), a high correlation between the Hilab system and the manual technique was observed.

Although these results demonstrate an expressive innovation in the point-of-care CBC field, we emphasize that this study presents some limitations. As a preliminary validation study, the interference of specific health conditions like icterus, dyslipidemias, neoplasms, and medicines interference on Hilab System results were not evaluated. In the same way, this study did not observe a significant number of blasts, metamyelocytes, and atypical lymphocytes, for example, to individually assess these kinds of cells. For these, more testing in different centers is in development.

## Conclusion

Our study provided an extensive clinical validation of the Hilab system to CBC point-of-care test, evaluating parameters like comparability, precision, and flagging studies. Over the entire measuring range, all values provided by this new approach presented high sensibility, specificity, and accuracy, compared to a sophisticated hematological analyzer (Sysmex XE-2100). Also, a high correlation was observed for all parameters evaluated. Thus, considering the need for blood count point-of-care tests, especially for quickly patient management, the study indicated that the Hilab system provides fast, accurate, low cost, and robust blood cell analysis for reliable clinical use. Considering the elevated costs associated with trained laboratory technicians, laboratory structure, and commercial hematological controls, besides the high time spent in individual blood smears analysis, the fast (average time to deliver the result to the patient: 40 min versus 24 h) and precise results of the Hilab device must be considered a relevant advantage of this hematological POCT.

## Supplementary Information

Supplementary Figure S1.

## Data Availability

The datasets used and/or analysed during the current study are available from the corresponding. Please contact alexia.gasparin@hilab.com.br.

## References

[1] Agarwal A, Bolosky WJ, Wilson DB (2019). Differentiation of leukemic blasts is not completely blocked in acute myeloid leukemia. Proc. Natl. Acad. Sci. U.S.A..

[2] World Health Organization (2020). The Selection and Use of Essential In Vitro Diagnostics.

[3] Celkan TT (2020). What does a hemogram say to us?. Turk. Pediatr. Ars..

[4] Bachar N, Benbassat D, Brailovsky D (2021). An artificial intelligence-assisted diagnostic platform for rapid near-patient hematology. Am. J. Hematol..

[5] Osei-Bimpong A, Jury C, Mclean R, Lewis SM (2009). Point-of-care method for total white cell count: An evaluation of the HemoCue WBC device. Int. J. Lab. Hematol..

[6] Yadav H, Shah D, Sayed S, Horton S, Schroeder LF (2021). Availability of essential diagnostics in ten low-income and middle-income countries: Results from national health facility surveys. Lancet Glob. Health.

[7] Kohli M, Walia K, Mazumdar S (2018). Availability of essential diagnostics in primary care in India. Lancet Infect. Dis.

[8] Wu AHB, Sellers J (2019). XW-100: First FDA CLIA-waived CBC analyzer designed for physician office use. J. Appl. Lab. Med..

[9] Rosenfeld LG, Malta DC, Szwarcwald CL (2019). Reference values for blood count laboratory tests in the Brazilian adult population, national health survey. Rev. Bras. Epidemiol..

[10] Ben-Yosef Y, Marom B, Hirshberg G (2016). The HemoScreen, a novel haematology analyser for the point of care. J. Clin. Pathol..

[11] Abbasi U, Chowdhury P, Subramaniam S (2019). A cartridge based Point-of-Care device for complete blood count. Sci. Rep..

[12] Rao LV, Ekberg BA, Connor D (2008). Evaluation of a new point of care automated complete blood count (CBC) analyzer in various clinical settings. Clin. Chim. Acta.

[13] Cable RG, Steele WR, Melmed RS (2012). The difference between fingerstick and venous hemoglobin and hematocrit varies by sex and iron stores. Transfusion.

