# Supplementary material for: Hilab system, a new point-of-care hematology analyzer supported by the Internet of Things and Artificial Intelligence
Source: Sci Rep. 2022 Jun 21;12:10409. doi: 10.1038/s41598-022-13913-8 (PMC9213419; doi:10.1038/s41598-022-13913-8)

**Legend of supplementary figures:**

**Figure S1 - Method comparison study between the Hilab System and the Sysmex XE-2100**. Mean (x̅), standard deviation (SD), and p-values of Paired Student T-test are demonstrated for each analyte.


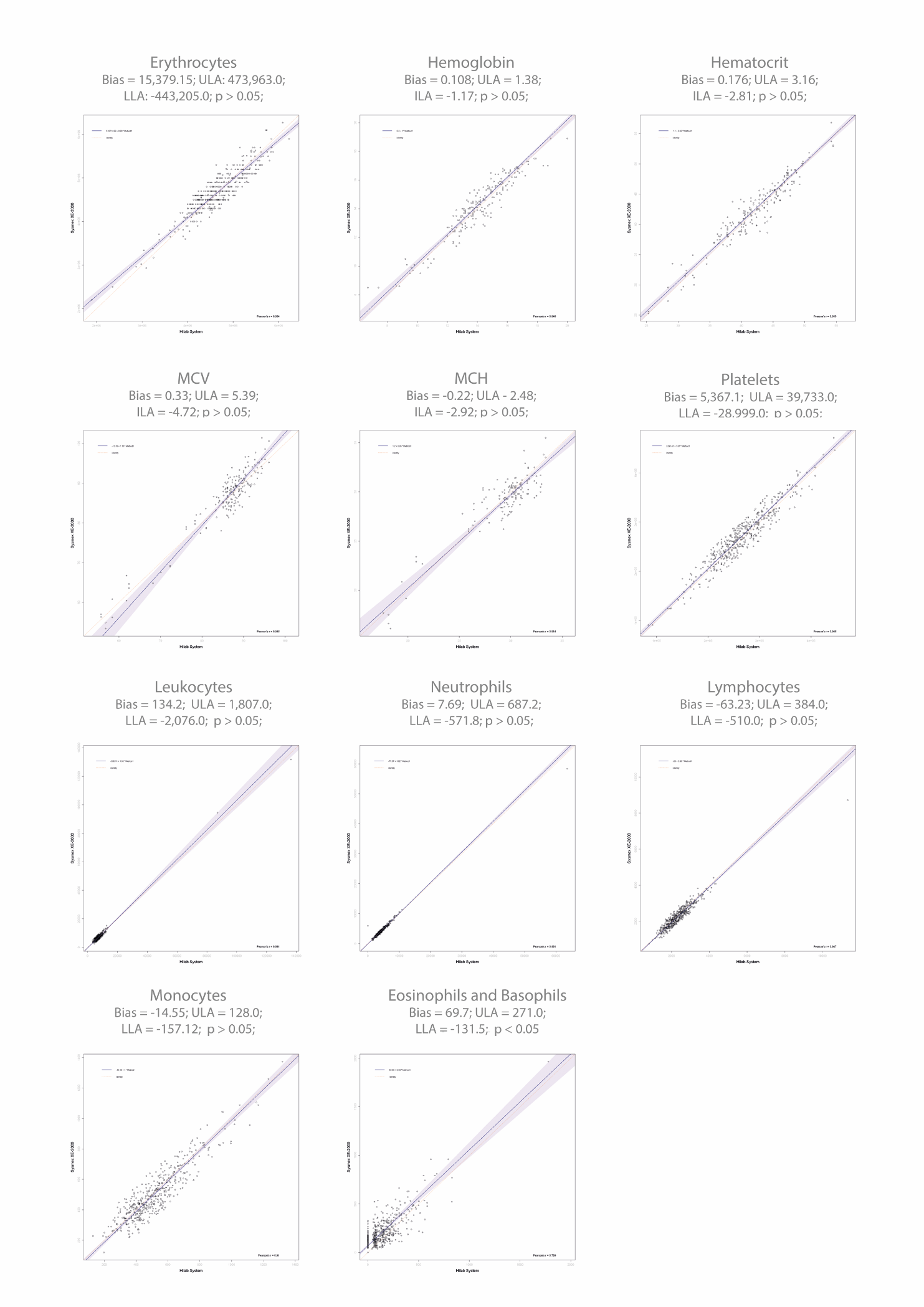

Supplement: Supplementary file 1 — Supplementary Figure S1. [file 41598_2022_13913_MOESM1_ESM.docx]
